# Gut Microbiota and Bacterial DNA Suppress Autoimmunity by Stimulating Regulatory B Cells in a Murine Model of Lupus

**DOI:** 10.3389/fimmu.2020.593353

**Published:** 2020-11-10

**Authors:** Qinghui Mu, Michael R. Edwards, Brianna K. Swartwout, Xavier Cabana Puig, Jiangdi Mao, Jing Zhu, Joe Grieco, Thomas E. Cecere, Meeta Prakash, Christopher M. Reilly, Christopher Puglisi, Prathyusha Bachali, Amrie C. Grammer, Peter E. Lipsky, Xin M. Luo

**Affiliations:** ^1^ Department of Biomedical Sciences and Pathobiology, Virginia-Maryland College of Veterinary Medicine, Virginia Tech, Blacksburg, VA, United States; ^2^ Translational Biology, Medicine and Health Graduate Program, Virginia Tech, Roanoke, VA, United States; ^3^ Carilion School of Medicine, Virginia Tech, Roanoke, VA, United States; ^4^ Edward Via College of Osteopathic Medicine, Blacksburg, VA, United States; ^5^ AMPEL BioSolutions LLC, Charlottesville, VA, United States

**Keywords:** systemic lupus erythematosus, immunoregulation, gut microbiota, autoimmunity, bacterial DNA

## Abstract

Autoimmune diseases, such as systemic lupus erythematosus, are characterized by excessive inflammation in response to self-antigens. Loss of appropriate immunoregulatory mechanisms contribute to disease exacerbation. We previously showed the suppressive effect of vancomycin treatment during the “active-disease” stage of lupus. In this study, we sought to understand the effect of the same treatment given before disease onset. To develop a model in which to test the regulatory role of the gut microbiota in modifying autoimmunity, we treated lupus-prone mice with vancomycin in the period before disease development (3–8 weeks of age). We found that administration of vancomycin to female MRL/lpr mice early, only during the pre-disease period but not from 3 to 15 weeks of age, led to disease exacerbation. Early vancomycin administration also reduced splenic regulatory B (Breg) cell numbers, as well as reduced circulating IL-10 and IL-35 in 8-week old mice. Further, we found that during the pre-disease period, administration of activated IL-10 producing Breg cells to mice treated with vancomycin suppressed lupus initiation, and that bacterial DNA from the gut microbiota was an inducer of Breg function. Oral gavage of bacterial DNA to mice treated with vancomycin increased Breg cells in the spleen and mesenteric lymph node at 8 weeks of age and reduced autoimmune disease severity at 15 weeks. This work suggests that a form of oral tolerance induced by bacterial DNA-mediated expansion of Breg cells suppress disease onset in the autoimmune-prone MRL/lpr mouse model. Future studies are warranted to further define the mechanism behind bacterial DNA promoting Breg cells.

## Background

Recent evidence suggests an association between the composition of gut microbiota and the pathogenesis of systemic lupus erythematosus (SLE), a systemic autoimmune disease affecting over 5 million people worldwide (1–3). We and others have reported intestinal dysbiosis in SLE patients and the abnormal dynamics of the gut microbiota in different lupus-prone mouse models (4–12). However, whether the gut microbiota is a cause or a secondary effect of lupus pathogenesis is still under debate. Moreover, whether the gut microbiota contribute only to the effector phase of SLE or play a more nuanced role in regulating the induction of SLE remains unknown. In female lupus-prone MRL/Mp-*Fas^lpr^* (MRL/lpr) mice, a significant depletion of *Lactobacilli* was observed (4). However, oral administration of a mixture of five *Lactobacillus* strains largely attenuated lupus-like symptoms in these mice (13), suggesting an essential role of the balance of microbiota genera in SLE pathogenesis. On the other hand, germ-free MRL/lpr female mice exhibited very similar lupus disease course and clinical parameters compared to mice housed under conventional conditions (14). This indicates that entire removal of gut microbiota throughout the lifespan neither attenuates nor exacerbates lupus. Rather, the effects of gut microbiota on lupus disease may be more complex and time-dependent, as we found that the removal of gut microbiota after lupus onset, achieved by treatment with mixed antibiotics (ampicillin, neomycin, metronidazole and vancomycin) or vancomycin alone, ameliorated lupus nephritis in female MRL/lpr mice (15). Whether there are other regulatory effects of the gut microbiota besides a role in the effector phase of disease, however, remains unresolved.

There is evidence that a variety of regulatory cells can modify lupus pathogenesis (16–18). Among these are regulatory B (Breg) cells that have been recognized as critical modulators of both normal and aberrant immune responses, especially in autoimmune disorders (19, 20). Numerical impairment of Breg cells has been observed in SLE patients, particularly those with active lupus nephritis (21). In mouse studies, the initial finding that B cell-deficient lupus-prone mice exhibited exacerbated disease brought the suppressive functions of Breg cells to light (22). Further studies revealed that the exacerbated disease phenotype was only seen when B cells were depleted early in life (23). In contrast, B cell depletion during late stages of disease was beneficial, consistent with the known function of B cells to produce pathogenic autoantibodies and present autoantigens to T cells in lupus (24, 25). This suggests that Breg-mediated protection from lupus may be restricted to the pre-disease stage. However, direct experimental evidence is lacking to support the hypothesis that the effect of Breg cells on lupus is time-dependent.

The involvement of the gut microbiota in Breg function has been of great interest in recent years (26, 27). Studies have linked microbiota to IL-10 producing Breg induction in colitis and arthritis mouse models, but the relationship between gut microbiota and Breg development in SLE has not been explored. We hypothesized that Breg cells could be induced by bacterial components, such as DNA, from the gut microbiota and that Breg function might suppress the development of SLE in disease-prone mice. Consistent with this, B cells isolated from lupus-prone mice produce more IL-10 in response to stimulation by CpG oligonucleotides than normal mouse B cells, but not to stimulation through the B-cell receptor or CD40 ligation (28). Moreover, B cells express toll-like receptor 9 (TLR9), the receptor of CpG-DNA; and TLR9-deficient lupus mice exhibit exacerbated disease suggesting a protective role for TLR9 ligation in lupus (29). These data support our hypothesis that bacterial DNA from the gut microbiota, rich in unmethylated CpG motifs (30), may promote the protective effects of Breg cells against lupus by inducing their IL-10 production.

Here, we develop a model in which to test the role of the gut microbiota in suppressing the initiation of SLE and present evidence for an important protective mechanism that involves bacterial DNA from the gut microbiota and the induction of Breg cells.

## Methods

### Mice and Treatments

Female MRL/Mp-*Fas^lpr^* (MRL/lpr, stock number 000485) mice were purchased from The Jackson Laboratory (Bar Harbor, ME) and bred and maintained in a specific pathogen-free facility according to the requirements of the Institutional Animal Care and Use Committee (IACUC) at Virginia Tech (Animal Welfare Assurance Number: A3208-01). CO_2_ was used for euthanasia according to the IACUC protocol. All experiments were performed in accordance with the National Institutes of Health guide for the care and use of Laboratory animals (NIH Publications No. 8023, revised 1978). Vancomycin (2 g/L) was given in the drinking water during the indicated periods of time. The drinking water containing vancomycin was replenished once a week. Endotoxin-free *E. coli* bacterial DNA was purchased from InvivoGen (San Diego, CA). Eighty micrograms per mouse of bacterial DNA was prepared in phosphate buffered saline (PBS) then orally gavaged to vancomycin-treated mice once a week for 4 consecutive weeks at 4, 5, 6, and 7 weeks of age. All experiments were performed at least twice.

### Renal Function

Urine was collected weekly starting from disease onset at 8 weeks of age, and all samples were stored at −20°C and analyzed at the same time with a Pierce Coomassie Protein Assay Kit (Thermo Scientific). When mice were euthanized at 15 weeks of age, kidneys were fixed in formalin for 24 h, embedded in paraffin, sectioned, and stained with periodic acid-Schiff (PAS) stain by Virginia Tech Animal Laboratory Services (ViTALs) at Virginia-Maryland College of Veterinary Medicine. Slides were read with an Olympus BX43 microscope. All the slides were scored in a blinded fashion by a board-certified veterinary pathologist. Glomerular lesions were graded on a scale of 0 to 3 for each of the following five categories: increased cellularity, increased mesangial matrix, necrosis, the percentage of sclerotic glomeruli, and the presence of crescents. Tubulointerstitial (TI) lesions were graded on a scale of 0 to 3 for each of the following four categories: presence of peritubular mononuclear infiltrates, tubular damage, interstitial fibrosis, and vasculitis.

### Cell Isolation and Flow Cytometry

Spleens were collected and mashed in 70-µm cell strainers with complete media (RPMI 1640, 10% fetal bovine serum, 1 mM sodium pyruvate, 1% 100× MEM non-essential amino acids, 10 mM HEPES, 55 μM 2-mercaptoethanol, 2 mM L-glutamine, 100 U/ml penicillin–streptomycin, all from Life Technologies, Grand Island, NY). Red blood cells were lysed with RBC lysis buffer (eBioscience). For surface staining, cells were blocked with anti-mouse CD16/32 (eBioscience), stained with fluorochrome-conjugated antibodies, and analyzed with BD FACS Aria II flow cytometer (BD Biosciences, San Jose, CA). For intracellular staining, Foxp3 Fixation/Permeabilization kit (eBioscience) was used. Zombie Aqua fixable viability kit (Biolegend) was used to exclude dead cells. Anti-mouse antibodies used in this study include the following: CD3-APC, CD4-PE-Cy7, CD8-PerCP/Cy5.5, IL-10-PerCP/Cy5.5, IFNγ-APC/Cy7, IL-17A-FITC, CD44-FITC, CD62L-APC/Cy7, Foxp3-PE, B220-PE, CD19-APC (Biolegend); RORγt-PE (eBioscience). Flow cytometry data were analyzed with FlowJo.

### RT-qPCR

Spleen and kidney were homogenized with Bullet Blender homogenizer (Next Advance), and total RNA was extracted with RNeasy Plus Universal Kit (Qiagen) according to the manufacturer’s instructions. Genomic DNA was removed by digestion with RNase free DNase I (Qiagen). Reverse transcription was performed by using iScript cDNA Synthesis Kit (Bio-Rad). Quantitative PCR was performed with iTaq Universal SYBR Green Supermix (Bio-Rad) and ABI 7500 Fast Real-Time PCR System (Applied Biosystems). Relative gene expression was calculated using *L32* as the housekeeping gene. Reactions were run in triplicate. Primer sequences for mouse *L32*, *IL-17A*, *Has1, Has2, Has3, IL-1β, TNFα* and *IL-6* are listed in **Supplemental Table I**.

### ELISA

Anti-dsDNA IgG was measured according to a previously described method (31). Relative anti-dsDNA IgG levels were calculated as the ratio between anti-dsDNA IgG (U/ml) and total IgG (mg/ml), and fold changes are shown with the ratio of the control group set as 1. Total IgG and cytokine concentrations were determined with mouse IgG (Bethyl Laboratories), IL-10, IL-6, IFN*γ* (Biolegend) and IL-35 (LifeSpan BioSciences) ELISA kits, respectively, according to the manufacturers’ instructions. Samples were run in duplicate.

### Breg Cell Isolation and Adoptive Transfer

IL-10 producing B cells were isolated from the spleens and mesenteric lymph nodes (MLNs) of sex- and age-matched (6 and 7 weeks of age, or 11 and 12 weeks of age) female MRL/lpr donor mice with the Mouse Regulatory B Cell Isolation Kit, purchased from Miltenyi Biotec (Gladbach, Germany) following the manufacturer’s protocol. Single cell suspensions were prepared from spleens and MLNs and B cells were enriched by using the Regulatory B cell Biotin-Antibody cocktail, followed by addition of Anti-Biotin MicroBeads and magnetic separation on LD Columns (Miltenyi Biotec). Regulatory B cells were enriched following *in vitro* stimulation of purified splenic B cells. Specifically, enriched B cells were stimulated for 16 h with 10 µg/ml lipopolysaccharide (LPS), followed by 5 h of 50 ng/ml phorbol myristate acetate (PMA) and 500 ng/ml ionomycin. Stimulated cells were labeled with Regulatory B Cell Catch Reagent (Miltenyi Biotec) and incubated for 45 min. The supernatant was removed, and cells were resuspended in buffer and labeled with Regulatory B Cell Detection Antibody. Anti-PE microbeads were mixed into the cell suspension and cells were magnetically separated on LS columns (Miltenyi Biotec) to capture IL-10 secreting Breg cells by positive selection. For adoptive transfer, each recipient mouse was injected with one million of these isolated Breg cells per injection through the tail vein. Two injections either at 6 and 7 weeks of age (pre-disease stage) or at 11 and 12 weeks of age (active-disease stage) were carried out. Mice in the control cell group were injected with cells depleted of Breg cells at 6 and 7 weeks of age. Specifically, the non-B cell fraction from the initial B-cell enrichment was combined with the negative fraction of stimulated B cells following IL-10 capture and magnetic isolation to constitute the control cell population used for adoptive transfer.

### Microbiota Sampling and Analyses

Fecal microbiota samples were obtained by taking a mouse out of the cage and collecting a fecal pellet. To avoid cross-contamination, each microbiota sample was collected by using a new pair of sterile tweezers. Samples were stored at −80°C. All samples were processed at the same time. Sample homogenization, cell lysis, and DNA extraction were performed as previously described (13). PCR was performed, and purified amplicons were sequenced bidirectionally on an Illumina MiSeq at Argonne National Laboratory. 16S rRNA gene sequencing data were analyzed as described previously (13). The datasets generated and analyzed during the current study are available in the NCBI SRA (BioProject #PRJNA529260). For gas chromatography measurement of short chain fatty acids (SCFAs), fecal samples were weighed then acidified using phosphoric acid immediately before analysis at Virginich Tech Environmental and Water Resources Research Facility. Quantification was performed with a Hewlett-Packard 5890 gas chromatograph fitted with a flame ionization detector coupled with a Nukol GC column and a Hewlett-Packard 7673 GC/SFC injector. The injector settings were: temperature -200°C; carrier-hydrogen; injection mode-split (ratio 2:1). The temperature program was: initial temperature 80°C held for 3 min, then increase temperature at a rate of 6°C per minute to 140°C and hold for 1 min. The Flame Ionization Detector Settings were: temperature-250°C; hydrogen flow-35 ml/min; air flow-350 ml/min; makeup flow (nitrogen)-15 ml/min; total makeup (makeup + column flow)-30 ml/min. The levels of individual SCFAs are shown as µmol/g feces.

### Oral Administration of SCFAs

MRL/lpr mice were treated with 2 g/L of vancomycin in the drinking water starting at 3 weeks of age. To avoid the possibility of chemical reactions between vancomycin and SCFAs in the drinking water, SFCAs were administered by oral gavage from 3 weeks of age to 8 weeks of age. Sodium acetate, sodium propionate and sodium butyrate (Sigma) were administered by oral gavage, at 1 g/kg of body weight, diluted in water, five times per week. Mice were then monitored during disease progression, and urine samples were collected weekly. Mice were sacrificed for sample collection at 15 weeks of age.

### Statistical Analysis

Analysis of non-sequencing data was performed with non-parametric Mann–Whitney test for the comparison of two groups, and non-parametric Kruskal–Wallis test for the comparison of three or more groups. Unless specified, the PBS-treated groups were used as the control. The results were considered statistically significant when *p* < 0.05. All analyses were performed with GraphPad Prism (San Diego, CA).

## Results

### Oral Vancomycin Given at the Pre-Disease Stage Exacerbates Lupus

**Figure 1:**
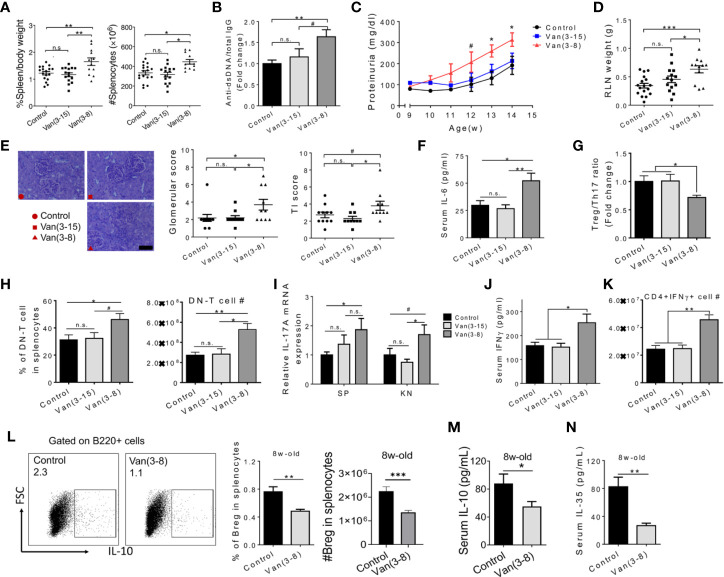
Oral vancomycin given at the pre-disease stage exacerbated lupus. Female MRL/lpr mice were given vancomycin (2 g/L) in the drinking water for the indicated time periods (in weeks). Analyses were performed at 15 weeks of age unless specified to be 8 weeks of age. **(A)** Spleen weight to body weight ratio (left) and total number of splenocytes (right). **(B)** The ratio of anti-dsDNA IgG to total IgG in the mouse serum (n ≥ 10). **(C)** Level of proteinuria over time (n ≥ 10). **(D)** RLN weight. **(E)** Renal histopathology. Left: representative PAS-stained kidney sections; bar equals 400 µm. Middle: glomerular score. Right: tubulointerstitial (TI) score. **(F)** Serum level of IL-6 (n ≥ 7). **(G)** Treg to Th17 ratio in the spleen (n ≥ 5). **(H)** Percentage and absolute number of DN-T cells in splenocytes (n ≥ 5). **(I)** Transcript level of *Il17a* gene in the spleen and kidney (n ≥ 7). **(J)** Serum level of IFN*γ* (n ≥ 5). **(K)** Absolute number of IFN*γ* producing CD4^+^ T cells in the spleen (n ≥ 5). **(L)** FACS analysis of IL-10^+^ Breg cells in the spleen at 8 weeks of age (n = 10). **(M)** Serum IL-10 at 8 weeks of age (n = 10). **(N)** Serum IL-35 at 8 weeks of age (n = 10). Data shown is representative of at least two independent experiments. ^#^ *p* < 0.1, *p < 0.05, **p < 0.01, ***p < 0.001. n.s., not statistically significant.

To explore the role of the microbiota in modulating the induction of SLE, we treated MRL/lpr mice with antibiotics before disease initiation. We chose vancomycin because it is not absorbed in the gastrointestinal tract (32), so any effect on the immune system should be mediated by local effects on the gut microbiota. As previously noted, both female (15) and male MRL/lpr mice (**Figures S1A–C**) benefited from oral vancomycin treatment during the active-disease stage, *i.e.*, 9 to 15 weeks of age. However, when we extended the time course of vancomycin treatment starting at 3 weeks and ending at 15 weeks of age, which covered both pre-disease and active-disease stages, the beneficial effects of vancomycin disappeared in both female (**Figures 1A–E**) and male MRL/lpr mice (**Figures S1A–C**). This is consistent with the lack of phenotype change between germ-free and specific pathogen-free MRL/lpr mice (14). We, therefore, hypothesized that the effects of gut microbiota on lupus was time-dependent, and questioned whether oral vancomycin treatment during the pre-disease stage, *i.e.*, 3 to 8 weeks of age, could promote lupus disease development. We decided to focus on female mice from then on as lupus has a strong female bias. Strikingly, splenomegaly in female MRL/lpr mice was significantly aggravated as a result of vancomycin treatment before disease onset (**Figure 1A**). In addition, the ratio of anti-double stranded (ds)DNA autoantibodies to total IgG in the serum was significantly elevated (**Figure 1B**). As kidney inflammation (or lupus nephritis) affects more than half of SLE patients (33), we assessed renal function by measuring proteinuria, renal lymph node (RLN) weight, and kidney histopathology. Both proteinuria and the RLN weight were significantly increased when mice were treated with oral vancomycin before disease onset (**Figures 1C, D**). Early vancomycin treatment also resulted in significantly higher pathologic scores in glomerular and tubulointerstitial (TI) evaluations (**Figure 1E**). These results indicate that gut microbiota has a time-dependent influence on the development of lupus, and that vancomycin-mediated modulation of gut microbiota before disease initiation could exacerbate lupus in female MRL/lpr mice.

To investigate the immunological mechanism(s) by which early vancomycin treatment worsened lupus, we first examined IL-6 and IL-17 as we had previously shown that diminished IL-6 and IL-17 levels contributed to the attenuated disease phenotype upon vancomycin treatment during the active-disease stage (15). We found that whereas long-term vancomycin treatment (3 to 15 weeks of age) did not change the serum level of IL-6, it was significantly upregulated in mice treated from 3 to 8 weeks only (**Figure 1F**). We next quantified splenic T-helper 17 (Th17) cells and CD4^−^CD8^−^ double-negative (DN) T cells, two major cellular sources of IL‑17 in both human and mouse lupus (34). A significant imbalance towards Th17 cells over regulatory T (Treg) cells was noted (**Figure 1G**). Moreover, the percentage and absolute number of DN T cells were significantly increased in mice treated with vancomycin before disease onset (**Figure 1H**). These data suggest a higher level of pro-inflammatory IL-17 cells as a result of treating mice with vancomycin before disease onset. Indeed, the transcript level of *Il17a* was significantly increased in both spleen and kidney (**Figure 1I**). The proportion of CD4^+^ and CD8^+^ T cells did not change (**Figures S1D, E**); however, an elevated level of IFN*γ*, which is known to promote lupus in both humans and MRL/lpr mice (35), was found in the serum of mice treated with vancomycin before disease onset (**Figure 1J**). Further analysis showed that oral vancomycin treatment before disease onset resulted in significantly more IFN*γ*-producing CD4^+^ T cells (or Th1 cells) in the spleen (**Figure 1K** and **Figure S1F**).

We next analyzed the phenotype of immune cells in 8-week-old mice right after vancomycin treatment before disease onset, and found a significantly reduced percentage of IL-10 producing Breg cells (**Figure 1L**), but not of Treg cells (**Figure S1G**). Breg cells produce large amounts of IL-10 and IL-35, two anti-inflammatory cytokines known as key mediators of the regulatory function of Breg cells (19). We found that treatment with vancomycin before disease onset significantly decreased the serum levels of both IL-10 and IL-35 at 8 weeks of age (**Figures 1M, N**), suggesting a functional loss of Breg cells after vancomycin treatment during the pre-disease stage. However, the percentage of Breg cells in 15-week-old mice did not differ (**Figure S1H**). This is likely related to the increase in Breg inducers during active disease, including self-DNA complexes and multiple elevated pro-inflammatory cytokines (26, 36). In addition, the bias towards DN T cells was already present at 8 weeks of age in mice treated with vancomycin (**Figure S1G**), suggesting early initiation of IL-17 production. Interestingly, administration of neomycin from 3 to 8 weeks of age led to minimal changes in RLN weight, proteinuria and circulating IL-6 (**Figure S2**), suggesting a vancomycin-specific effect on exacerbation of lupus nephritis. Neomycin, a systemically absorbed antibiotic, might have additional effects apart from its action on the gut microbiota. Notably, anti-dsDNA autoantibodies were increased with neomycin (**Figure S2B**) but did not seem to play a pathogenic role. The implications of this observation are unclear.

Taken together, these results suggest that vancomycin-mediated modulation of gut microbiota before disease onset significantly reduced Breg cells, resulting in systemic inflammation that may contribute to lupus exacerbation in female MRL/lpr mice.

### Adoptive Transfer of Breg Cells Before Disease Onset Attenuates Lupus in Vancomycin-Treated Mice

**Figure 2:**
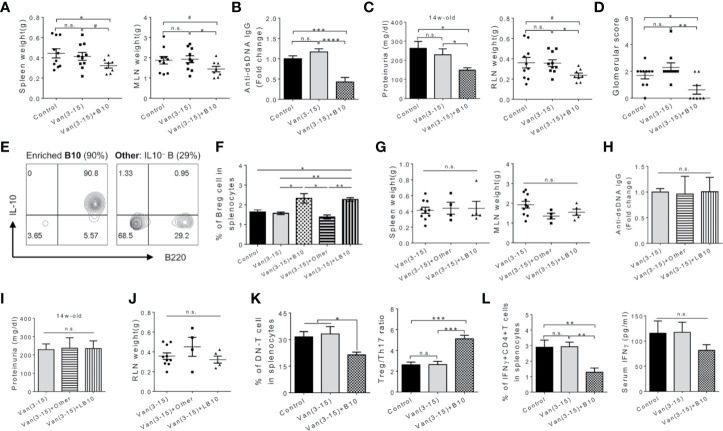
Adoptive transfer of Breg cells at the pre-disease stage attenuated lupus in vancomycin-treated mice. Female MRL/lpr mice were treated with vancomycin (2 g/L) from 3 to 15 weeks of age. Adoptive transfer of cells (10^6^/mouse per dose) were performed at indicated times. Analyses were performed at 15 weeks of age. B10, early injection of activated IL-10^+^ Breg cells at 6 and 7 weeks of age. **(A)** Spleen and MLN weight. **(B)** Anti-dsDNA IgG level in the mouse serum (n ≥ 8). **(C)** Proteinuria level (left; n ≥ 8) and renal lymph node (RLN) weight (right). **(D)** Kidney glomerular score. **(E)** FACS plot showing enriched IL-10^+^ Breg cells and composition of the control cell population. **(F)** Percentage of IL-10 producing B cells in the spleen (n ≥ 7). The negative controls are adoptive transfer of non-Breg cells (Other) at 6 and 7 weeks of age, and late administration of activated IL-10^+^ Breg cells (LB10) at 11 and 12 weeks of age. **(G)** Spleen and MLN weight. **(H)** Anti-dsDNA IgG level in the serum (n ≥ 4). **(I)** Proteinuria level (n ≥ 4). **(J)** RLN weight. **(K)** Percentage of DN T cells (left) and Treg to Th17 ratio (right) in the mouse spleen (n ≥ 5). **(L)** Percentage of IFNγ producing CD4^+^ T cells in the mouse spleen (left) and serum IFN*γ* (right) (n ≥ 5). Data shown is representative of at least two independent experiments. #*p* < 0.1, *p < 0.05, **p < 0.01, ***p < 0.001, ****p < 0.0001. n.s., not statistically significant.

To directly address the hypothesis that Breg cells served to protect mice from developing SLE, we adoptively transferred Breg cells into vancomycin-treated mice at different disease stages. Compared to untreated mice and mice treated with vancomycin only, early transfer of enriched IL-10 producing Breg cells (90% purity) at 6 and 7 weeks of age significantly reduced weights of secondary lymphoid organs (**Figure 2A**) and the level of anti-dsDNA autoantibodies at 15 weeks of age (**Figure 2B**). In addition, adoptive transfer of Breg cells before disease onset significantly ameliorated lupus nephritis with reduced proteinuria, smaller RLN and less severe glomerular pathology (**Figures 2C, D**). Importantly, transfer of cells depleted of IL-10 producing Breg cells, which included 29% activated IL-10^−^ B cells and 70% non-B cells (**Figure 2E**) but no introduction of Breg cells (**Figures 2E, F**), during the same time frame did not have the protective effects (**Figures 2G–J**). Furthermore, although Breg cells were increased in number regardless of the time of transfer (**Figure 2F**), Breg cells injected during the active-disease stage were not beneficial (**Figures 2G–J**). The lack of beneficial response to late Breg injection suggests that the immunosuppressive function of Breg cells may be more pronounced during the pre-disease stage of lupus development.

We next examined immune cell populations and cytokine production with or without adoptive transfer of Breg cells. Breg cell transfer during the pre-disease stage significantly decreased the percentage of DN T cells and altered the balance of Treg/Th17 towards Treg cells (**Figure 2K** and **Figure S2E**). Additionally, the percentage of IFN*γ*-producing CD4^+^ T (or Th1) cells significantly decreased in mice receiving Breg cells before disease onset (**Figure 2L** and **Figure S2F**). The level of IFN*γ* in the circulation was not significantly affected, although there was a trend for decline. These results suggest that increasing the abundance of activated Breg cells in the vancomycin-treated mice—where Breg function was impaired—reduced IL-17 and IFN*γ* producing cells leading to attenuation of systemic manifestations and prevention of the progression of nephritis in MRL/lpr mice. Whether activated Breg cells mitigated lupus in a IL-10 dependent manner, or through the production of other cytokines such as IL-35, will be investigated in the future.

### Disease Stage-Specific Effects of Oral Vancomycin on the Gut Microbiota

**Figure 3:**
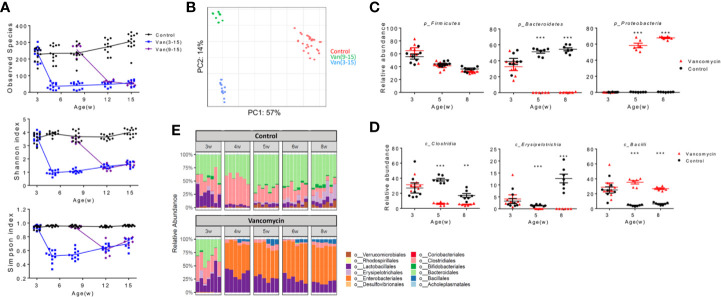
Disease stage-specific effects of oral vancomycin on the gut microbiota. Fecal samples were collected at indicated times and analyzed with 16S rRNA sequencing. **(A)** Diversity and richness of gut microbiota at different ages. Top: Observed species; Middle: Shannon index; Bottom: Simpson index (n ≥ 6). **(B)** Weighted PCA analysis of gut microbiota at 15 weeks of age (n ≥ 10). **(C)** Relative abundance of phylum Firmicutes, Bacteroidetes and Proteobacteria at 3, 5, and 8 weeks of age (n ≥ 6). **(D)** Relative abundance of class Clostridia, Erysipelotrichia and Bacilli at 3, 5, and 8 weeks of age (n ≥ 6). **(E)** Relative abundance at the order level from 3 to 8 weeks of age (n ≥ 6). qPCR data **(C, D)** are representative of at least two independent experiments. **p < 0.01, ***p < 0.001.

The components of the gut microbiota removed by oral vancomycin may be responsible for inducing Breg cells at the pre-disease stage before disease onset in MRL/lpr mice. Analysis of gut microbiota revealed that the diversity and richness of commensal bacteria were quickly—within 2–3 weeks after oral vancomycin was started—and significantly reduced to similar levels regardless of the treatment time frame (**Figure 3A**). However, the composition of the gut microbiota at 15 weeks of age was quite different between the two vancomycin-treated groups (**Figure 3B**), indicating that the same antibiotic could result in different changes of gut microbiota when given at different times. Focusing on the pre-disease period where vancomycin exacerbated autoimmunity, we found significant changes in the gut microbiota at several taxonomical levels. At the phylum level, in vancomycin-treated young mice, Bacteroidetes were completely removed and replaced by Proteobacteria whereas Firmicutes remained unchanged (**Figure 3C**). Within the phylum Firmicutes, changes in multiple classes were observed, specifically, the decline of Clostridia and Erysipelotrichia but increase of Bacilli (**Figure 3D**). Within the class Clostridia, every major family decreased, including *Clostridales* and *Lachnospiraceae* (**Figure S3A**). Interestingly, within the class Bacilli, whereas the order Lactobacillales was more abundant with vancomycin treatment (**Figure 3E**), not every *Lactobacillus* species increased its abundance: *L. animalis* flourished, *L. intestinalis* decreased, whereas *L. frumenti* remained unchanged (**Figure S3B**). At the order level, the major alterations were expansions of Lactobacillales and Enterobacteriales along with complete removal of Bacteroidales and Clostridiales (**Figure 3E**).

### Restoration of Bacterial DNA Attenuates Lupus in Vancomycin-Treated Mice

As oral vancomycin can remove many producers of short chain fatty acids (SCFAs) (37, 38), we quantified the levels of fecal SCFAs with gas chromatography. In female MRL/lpr mice, the level of total SCFAs in the feces increased from 3 to 5 weeks of age then plateaued (**Figure S4A**). Vancomycin significantly lowered the level of fecal SCFAs, consistent with vancomycin-mediated removal of Clostridia (**Figure S4B**). Further analysis showed that the three most abundant SCFAs—acetate, propionate and butyrate—were all significantly reduced in the feces with vancomycin treatment (**Figure S4C**). In contrast, the fecal heptanoate level significantly increased although its role in immune regulation and autoimmunity is unknown. In light of the known effect of SCFAs on Treg cells (39), we examined whether restoration of SCFAs could promote Breg cells and attenuate lupus in vancomycin-treated mice. Our result showed that supplementation with SCFAs did not increase the percentage of Breg cells (**Figure S4D**). Consequently, SCFA supplement did not attenuate either systemic autoimmunity (**Figure S4E**) or lupus nephritis (**Figure S4F**). These results suggest that the reduction of SCFAs upon vancomycin administration was not the reason for Breg impairment and disease exacerbation.

**Figure 4:**
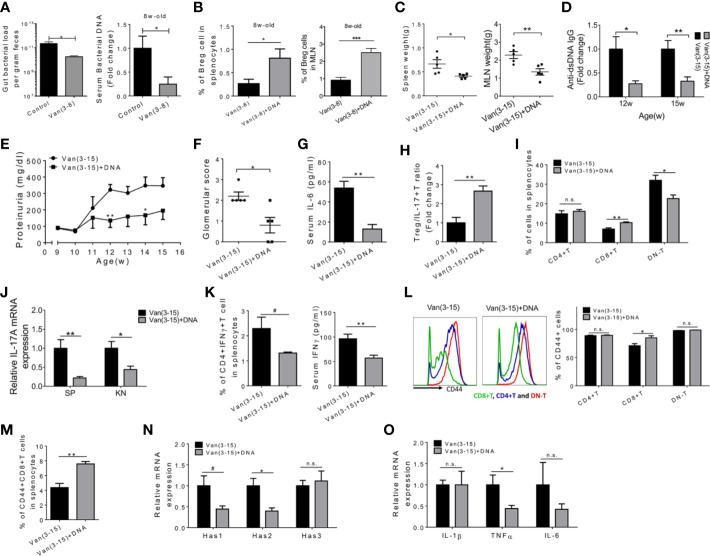
Restoration of bacterial DNA attenuated lupus in vancomycin-treated mice. Female MRL/lpr mice were treated with vancomycin (2 g/L) starting 3 weeks of age. Endotoxin-free *E. coli* bacterial DNA (80 µg in PBS/mouse per dose) was orally gavaged to vancomycin-treated mice once a week for 4 consecutive weeks at 4, 5, 6, and 7 weeks of age. Analyses were performed at 15 weeks of age unless specified. The vancomycin-treated group was used as the control group except for panel A. **(A)** Left: bacterial DNA load in the feces at 7 weeks of age (n = 9). Right: bacterial DNA load in the circulation at 8 weeks of age (n = 5). **(B)** Percentage of IL-10 producing Breg cells in the spleen and MLN at 8 weeks of age (n = 5). **(C)** Spleen and MLN weight. **(D)** Serum anti-dsDNA IgG level at 12 and 15 weeks of age (n = 5). **(E)** Level of proteinuria over time (n = 5). **(F)** Kidney glomerular score. **(G)** Serum level of IL-6 (n = 5). **(H)** The ratio of Treg to Th17 cells in the spleen (n = 5). **(I)** The percentages of CD4^+^, CD8^+^ and DN T cells in the spleen (n = 5). **(J)** Transcript level of *Il17a* gene in the spleen and kidney (n = 5). **(K)** The percentage of IFN*γ* producing CD4^+^ T cells in the spleen (left) and IFN*γ* level in the serum (n = 5). **(L)** Representative CD44 FACS histogram (left) and percentage of CD44^+^ cells in CD4^+^, CD8^+^ and DN T cells (right) (n = 5). **(M)** Percentage of CD44^+^CD8^+^ T cells in the spleen (n = 5). **(N)** Transcript level of *Has1*, *Has2*, and *Has3* genes in the kidney (n = 5). **(O)** Transcript level of *IL1β*, *TNFα*, and *IL6* genes in the kidney (n = 5). #*p* < 0.1, *p < 0.05, **p < 0.01, ***p < 0.001. n.s., not statistically significant.

Through removal of gut microbiota, vancomycin would also decrease the bacterial load and thus bacteria-derived DNA, the latter of which had been shown to suppress lupus in mice (40). Interestingly, in our mice, vancomycin led to a significant decrease in the total bacterial load in the gut and a significant reduction of bacterial DNA in the circulation (**Figure 4A**). We therefore asked whether bacterial DNA could be the Breg inducer that would protect vancomycin-treated mice from disease exacerbation. Notably, recent studies have shown a role of oral antigens in the induction of tolerance involving Breg cells (41–46), but previous studies have not shown a role of orally administered nucleic acids in tolerance. We, therefore, sought to determine whether orally gavaged endotoxin-free *E. coli* DNA, administered once a week from 4 to 7 weeks of age at 80 µg/dose, would induce Breg-mediated oral tolerance in vancomycin-treated female MRL/lpr mice. Strikingly, oral administration of bacterial DNA led to a significant increase of Breg cells in the spleen and MLN (**Figures 4B** and **S5A, B**) without affecting Foxp3^+^ Treg cells, Foxp3^+^ROR*γ*t^+^ Treg cells, or IL-10 producing CD4^+^ T cells (**Figures S5C, D**). As a result, in vancomycin-treated mice given oral bacterial DNA, the spleen and MLN weights (**Figure 4C**), serum level of anti-dsDNA autoantibodies (**Figure 4D**), proteinuria (**Figure 4E**), and kidney glomerular score (**Figure 4F**) all significantly decreased. These results suggest that orally administered bacterial DNA can induce a form of oral tolerance and be protective against lupus progression, and that the reduction in circulating bacterial DNA by vancomycin treatment during the pre-disease stage may be the cause of lupus exacerbation.

We next investigated the effects of bacterial DNA on inflammatory mediators in the circulation and their cellular sources in the spleen. Serum IL-6 was significantly decreased by oral DNA treatment (**Figure 4G**). Correspondingly, the ratio of Treg cells to Th17 cells was significantly increased in the spleen (**Figures 4H** and **S5E**). DN T cells, another major IL-17 producer, were significantly reduced, together with a significant increase of the proportion of CD8^+^ T cells (**Figure 4I**). In both spleen and kidney, the expression of *Il17a* mRNA was significantly decreased following bacterial DNA treatment (**Figure 4J**). In addition, IFN*γ* production from splenic CD4^+^ T cells was inhibited, resulting in a significant decrease of serum IFN*γ* (**Figure 4K**). Moreover, the expression of CD44 in CD8^+^ T cells was significantly elevated in the spleen of bacterial DNA-treated mice (**Figure 4L**), resulting in a significantly higher percentage of CD44^+^CD8^+^ T cells (**Figure 4M**). CD44 is important for T cell migration into inflammatory sites such as the nephritic kidney (47). Because of their potential protective effect against murine lupus (48–50), more CD8^+^ T cells infiltrating the kidney may be beneficial. Furthermore, treatment with bacterial DNA significantly decreased the transcript levels of type 1 and type 2 hyaluronan synthases (*Has*) in the kidney (**Figure 4N**), enzymes that mediate the secretion of hyaluronate (HA) (51). HA is known as the principal extracellular CD44 ligand (51). CD44–HA interaction mediates the recruitment of diverse immune cells, in particular T cells, and contributes to disease activity in lupus (52–54). In patients and mice with active lupus nephritis, the secretion of HA is enhanced in kidney, which correlates with lymphocyte infiltration and kidney damage (55, 56). Importantly, the inhibition of HA in lupus-prone mice improved disease parameters, at least partially by reducing the expression of pro-inflammatory cytokines, including IL-1β, TNFα, and IL-6, in the kidney (57). We noted that the expression of TNFα mRNA in the kidney was also significantly reduced (**Figure 4O**). This may be another mechanism by which the administration of bacterial DNA attenuated lupus nephritis in vancomycin-treated female MRL/lpr mice. Notably, the administration of bacterial DNA to mice treated with vancomycin did not significantly alter the composition of the small intestine microbiota (**Figure S6**), suggesting that bacterial DNA may have bypassed the gut microbiota to induce tolerance in these mice.

## Discussion

B cells can promote autoimmunity because of their capabilities to produce autoantibodies and stimulate autoreactive T cells (58). However, a subpopulation of B cells, namely Breg cells, are immunosuppressive (19). Defective IL-10 production and reduced immunosuppressive ability have been observed in Breg cells isolated from the peripheral blood of SLE patients (59). This indicates impairment of the regulatory function of Breg cells in human SLE, highlighting them as a potential therapeutic target. Unlike Treg cells that express *Foxp3*, Breg cells do not have a common transcription factor. Any B cells may differentiate into IL-10 producing Breg cells in response to the proper stimulation (19). The gut microbiota has been suggested as an important environmental stimulus of Breg cells as wildtype mice treated with mixed antibiotics had fewer Breg cells (26), a phenomenon consistent with our observation in lupus-prone mice.

To study the role of the gut microbiota on Breg cells, we chose the antibiotic vancomycin as it is not absorbed in the gut (32). Antibiotics readily absorbed in the intestine, such as neomycin, might have systemic effects beyond those on the gut microbiota. In this study, we found that vancomycin treatment before disease onset significantly removed bacterial DNA from the gut and circulation. In parallel, the development of Breg cells at 8 weeks of age was dramatically inhibited, leading to more severe clinical outcomes. Importantly, restoration of bacterial DNA in the antibiotic-treated mice increased the abundance of Breg cells in the spleen and MLN at 8 weeks of age, downregulated IL-17 and IFN*γ* related immune responses, and significantly attenuated lupus. These results are consistent with those of an earlier study in NZB/W F1 mice, where *i.p.* immunization with bacterial DNA was protective against lupus nephritis (40). However, no mechanism was given in the earlier study, and the administration route of bacterial DNA was different (*i.p.* at the dose of 50 µg). We administered bacterial DNA orally to mimic the contribution of the gut microbiota and to determine a role of bacterial DNA in inducing a form of oral tolerance that mitigated autoimmunity. Future studies involving conditional knockout of DNA sensors will delineate the mechanism by which bacterial DNA induces Breg cells to protect against lupus. Notably, a recent study has shown that B cell-specific expression of TLR9, a sensor for bacterial DNA, is protective against lupus (60).

NZB/W F1 mice deficient of CD19, compared to unaltered NZB/W F1 mice, showed an earlier onset of lupus nephritis and exhibited a reduced survival rate, though the emergence of autoantibodies was also delayed (22). In another study, the depletion of all mature B cells, including Breg cells, accelerated disease onset (23). However, the exacerbation of lupus was only seen in NZB/W F1 mice with B-cell depletion initiated very early in life from 4 weeks of age. In contrast, when B-cell depletion started at 12 to 28 weeks of age, the disease was significantly inhibited. It is likely that early depletion of B cells largely removed Breg cells, whereas late depletion was likely to have eliminated more autoreactive B cells. While using a different lupus-prone mouse model, we observed a very similar phenomenon on the development and effect of Breg cells. Early reduction of circulating bacterial DNA by vancomycin treatment significantly decreased the percentage of Breg cells in the spleen, resulting in higher levels of IFN*γ* production, IL-17 mRNA expression, earlier disease initiation, and exacerbation of lupus. On the contrary, the same antibiotic dosage given at a later time (9 to 15 weeks of age) attenuated lupus-like disease in the same mouse model (15). Interestingly, replenishment of bacterial DNA from either gram-positive or gram-negative bacteria, through oral gavage, increased the Breg cells in both spleen and MLN at 8 weeks of age, while reducing IFN*γ*, TNFα, and late-stage disease severity (data not shown). Additional supporting evidence for the role of Breg cells in delaying disease initiation and late-stage disease severity is that adoptive transfer of activated Breg cells ameliorated disease parameters in vancomycin-treated mice when injected pre-disease, while no reduction in disease severity was observed in mice administered activated Breg cells following autoimmune disease development. These results clearly indicate that the immunosuppressive functions of Breg cells act to suppress initiation of autoimmune disease. Notably, we had used a mixture of IL-10^-^ B cells (29%) and non-B cells (70%) as the cell control that also included T cells such as Treg cells. This cell control given at the pre-disease stage did not change the disease severity at 15 weeks of age, suggesting that early introduction of activated IL-10 producing Breg cells, rather than Treg cells, were responsible for the attenuation of lupus. In addition, we found that the percentages of Breg cells in the spleen were similar at the late-disease stage (15 week of age) regardless of treatment. This has been shown to be related to the accumulation of DNA-containing immune complexes during active disease, which, together with elevated pro-inflammatory cytokines, can dominate the regulatory activity of Breg cells (36). This may explain why antibiotic treatment during the active-disease stage is beneficial, even with the removal of bacterial DNA.

In this study, we have presented a novel mechanism in which commensal microbiota are able to promote Breg abundance and function during early autoimmune disease. It is evident that bacterial DNA can induce Breg cells during vancomycin treatment, promoting immunosuppressive cytokine production and mitigating disease exacerbation. Moreover, other cells may contribute to Breg activation, following bacterial DNA recognition, through cytokine production or antigen presentation, as shown by the ability of plasmacytoid dendritic cell-derived Type I IFNs to modulate TLR7 sensitivity of naïve B cells (61). Additional studies are needed to define the underlying mechanism of bacterial DNA promoting immunoregulation more completely. Notably, however, while several oral antigens have been shown to induce Breg cells (41–46), the role of oral nucleic acids in tolerance induction has not been reported. We show here that bacterial DNA is capable of inducing a form of oral tolerance that mitigates autoimmunity in lupus mice.

In MRL/lpr mice, the results are somewhat controversial regarding the role of Breg cells. One report described Breg cells as protective, as the transfer of *in vitro* anti-CD40-generated B cells greatly improved lupus nephritis through an IL-10-dependent mechanism (62). However, in another study, B-cell-specific IL-10 deletion did not affect lupus progression, implying that endogenous IL-10 producing Breg cells are ineffective in suppressing autoimmunity in MRL/lpr mice (63). This may be explained by the time dependence of Breg function, and an inducible model to deplete IL-10 producing Breg cells at different time points will help delineate the importance of these cells at different disease stages. Moreover, it is likely that other anti-inflammatory mediators originated from Breg cells can compensate the loss of IL-10 in regulating autoimmunity. Indeed, IL-10 independent immune suppression by Breg cells also occur (19). For example, a unique subset of IL-35 producing B cells overlaps with the IL-10^+^ Breg subset. The promotion of IL-35^+^ Breg cells *in vivo* conferred protection against autoimmune disease (64). In our study, the reductions of IL-10^+^ Breg cells and serum IL-10 were accompanied by reduced serum IL-35, suggesting that IL-35 may be responsible for the regulatory function of Breg cells on lupus in MRL/lpr mice. It is unknown if this reduction in IL-35 is primarily related to decreased Breg cell induction and reduced Breg IL-35 production, or reduced IL-35 production from regulatory CD4^+^ or CD8^+^ T cells subsequently leading to reduced Breg induction. Since IL-35 can induce IL-10 producing Breg cells, it is possible that the reduced circulating IL-35 observed with early vancomycin treatment contributes to the reduced Breg induction. As such, a role for Treg cells or an underlying metabolic mechanism contributing to the reduced Breg abundance and function observed following early vancomycin treatment cannot be ruled out. Further studies involving B cell-specific deficiency of IL-35, preferably in an inducible model, will help to elucidate the protective role of IL-35 producing Breg cells against lupus.

In summary, vancomycin administered prior to autoimmune phenotype development exacerbates disease markers, in part, through reduced Breg cell numbers and circulating Breg-associated cytokines. Replenishment of IL-10 secreting Breg cells through adoptive transfer reduced autoimmune disease severity in 15 week-old female MRL/lpr mice. In addition, oral administration of bacterial DNA induced Breg cells and attenuated lupus. This work highlights a novel pathway of immune-regulatory modulation by gut microbiota-supplied DNA that acts through promotion of Breg cells in secondary lymphoid organs to restrain the development of autoimmune disease.

## Data Availability Statement

The datasets presented in this study can be found in online repositories. The names of the repository/repositories and accession number(s) can be found below: https://www.ncbi.nlm.nih.gov/, BioProject #PRJNA529260.

## Ethics Statement

The animal study was reviewed and approved by Institutional Animal Care and Use Committee (IACUC) at Virginia Tech (Animal Welfare Assurance Number: A3208-01).

## Author Contributions

This study was conceived and designed by QM and XL. The experiments were performed by QM, ME, BS, XP, JM, JZ, JG, MP, CP, and PB. Histopathological scoring was performed by TC. Data analysis was performed by QM, ME, and XL. The manuscript was composed by QM, ME, CR, AG, PL, and XL. All authors contributed to the article and approved the submitted version.

## Funding

NIAMS AR073240, AR067418 (XL).

## Conflict of Interest

PL, AG, CP, and PB are employed by the company AMPEL BioSolutions LLC, Charlottesville, VA.

The remaining authors declare that the research was conducted in the absence of any commercial or financial relationships that could be construed as a potential conflict of interest.
