# Supplementary material for: Gut Microbiota and Bacterial DNA Suppress Autoimmunity by Stimulating Regulatory B Cells in a Murine Model of Lupus
Source: Front Immunol. 2020 Nov 10;11:593353. doi: 10.3389/fimmu.2020.593353 (PMC7683516; doi:10.3389/fimmu.2020.593353)
Supplement: Supplementary file 1 [file DataSheet_1.pdf]

Supplementary Figure 1

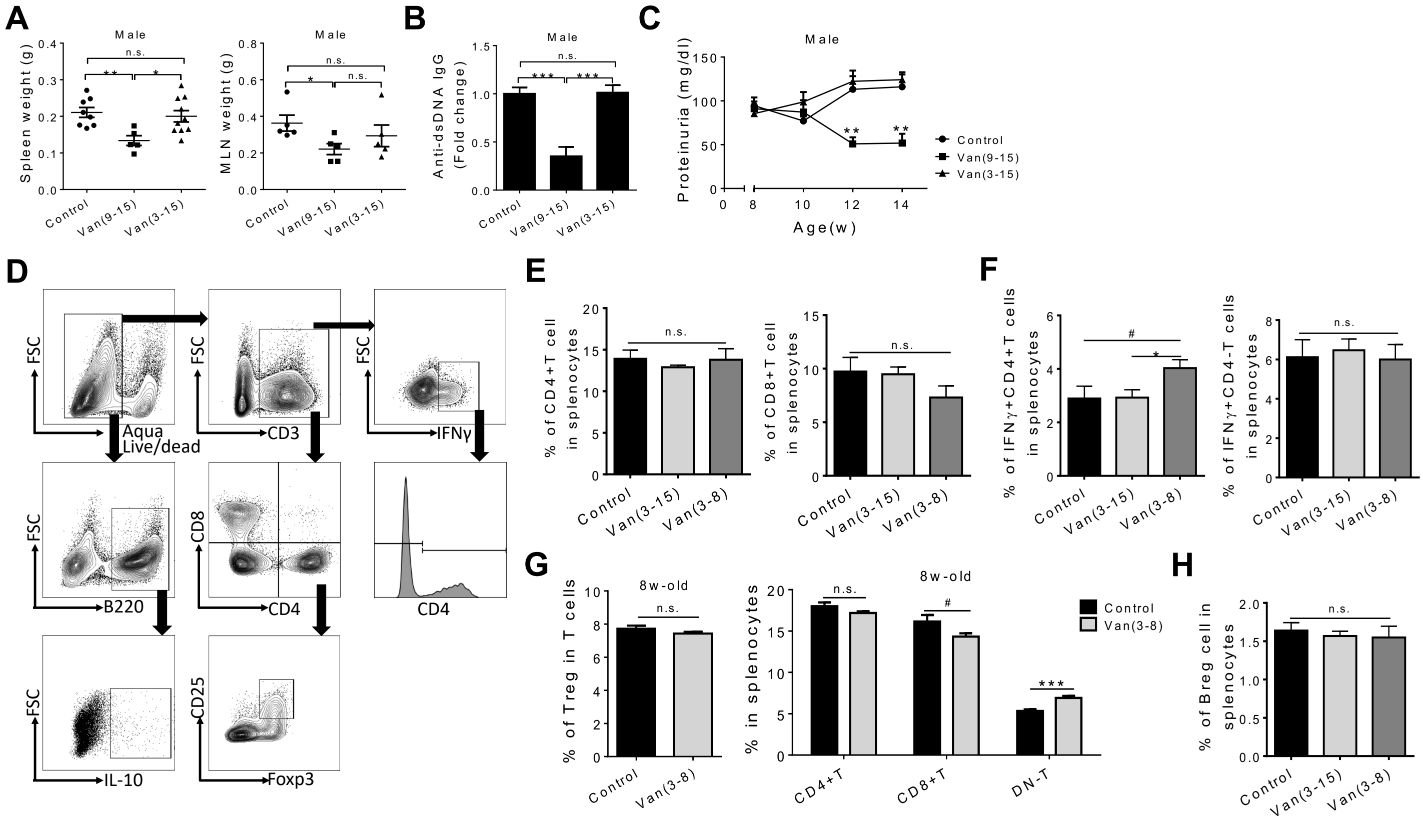

Figure S1. (A) Spleen and MLN weight at 15 weeks of age. (B) Anti-dsDNA IgG level in the mouse serum at 15 weeks of age (n≥5). (C) Level of proteinuria over time (n≥5). (D) Gating strategy for the FACS analysis of IL-10$^+$ Breg, IFNγ$^+$ T cells, DN T cells, and CD25$^+$Foxp3$^+$ Treg cells. (E) The percentage of CD4$^+$ and CD8$^+$ T cells in the spleen at 15 weeks of age (n≥5). (F) The percentage of IFNγ producing CD4$^+$ and CD4$^-$ T cells in the spleen at 15 weeks of age (n≥5). (G) The percentage of Treg cells (left) and CD4$^+$, CD8$^+$ and DN T cells (right) in the spleen at 8 weeks of age (n=10). (H) The percentage of IL-10$^+$ Breg cells in the spleen at 15 weeks of age (n≥5). #*p*<0.1, **p*<0.05, ***p*<0.01, ****p*<0.001. n.s., not statistically significant.

Supplementary Figure 2

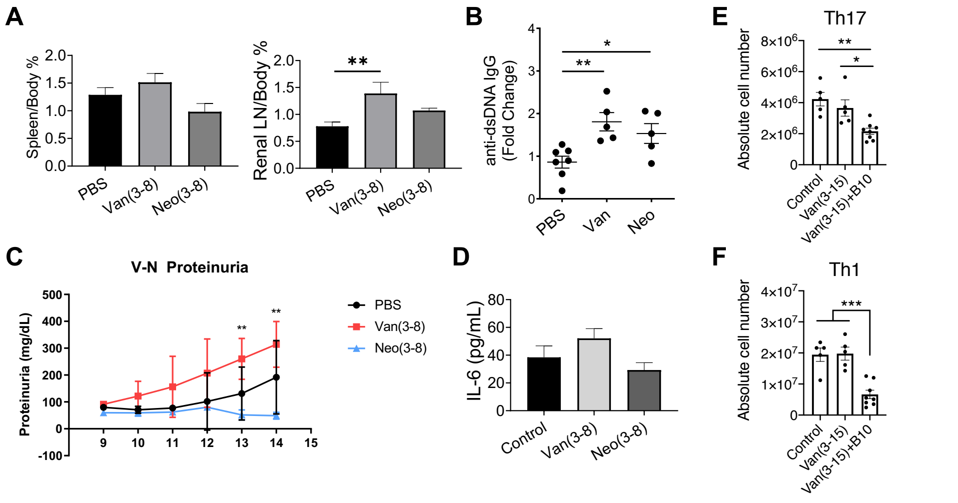

Figure S2. (A) Percent of spleen weight and RLN weight as compared to body weight at 15 weeks of age (n≥8). (B) Serum anti-dsDNA IgG autoantibody levels at 15 weeks of age. (C) Level of proteinuria from 9-14 weeks of age. (D) Serum IL-6 concentration (n≥5). (E) Absolute number of Th17 cells in the spleen at 15 weeks of age. (F) Absolute number of Th1 cells in the spleen at 15 weeks of age. *p<0.05, **p<0.01, ***p<0.001.

Supplementary Figure 3

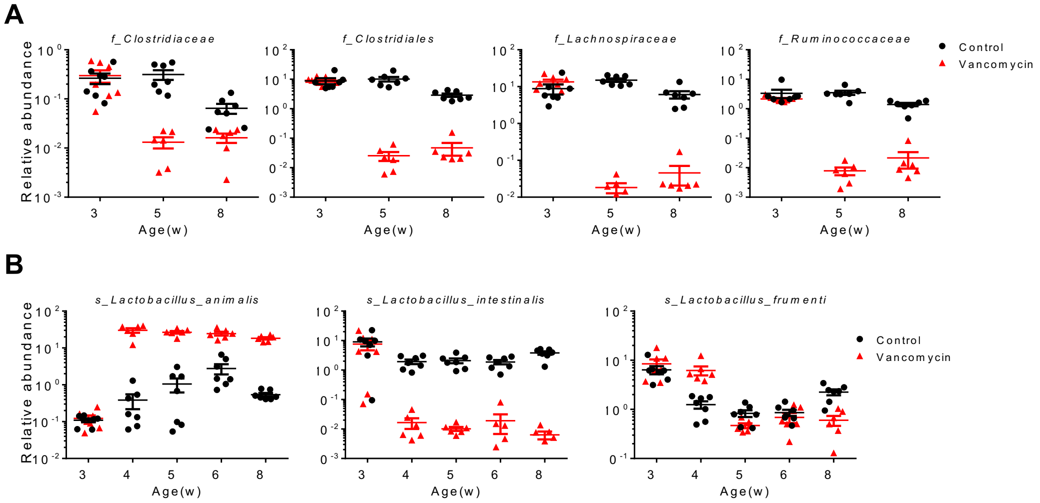

Figure S3. (A) Relative abundance of family *Clostridiaceae*, *Clostridiales*, *Lachnospiraceae* and *Ruminococcaceae*. (M) Relative abundance of species *L. animalis, L. intestinalis* and *L. frumenti*.

Supplementary Figure 4

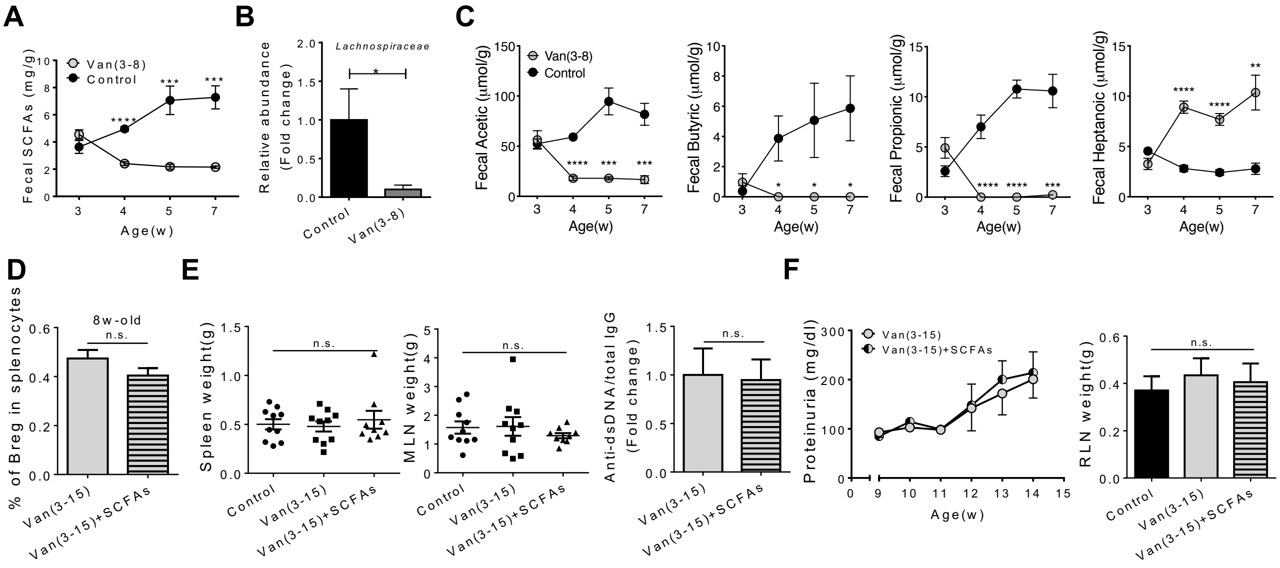

Figure S4. (A) Level of total SCFAs in the feces over time (n≥4). (B) Relative abundance of *Lachnospiraceae* in the feces at 7 weeks of age (n=9). (C) Levels of acetic, butyric, propionic and heptanoic acids in the feces over time (n≥4). (D) Percentage of Breg cells in the spleen at 8 weeks of age (n=5). (E) Spleen (left) and MLN (middle) at 15 weeks of age, and anti-dsDNA IgG to total IgG ratio (right) in the serum at 12 weeks of age (n≥9). (F) Level of proteinuria over time (left) and RLN weight (right) (n≥9). *p<0.05, **p<0.01, ***p<0.001. n.s., not statistically significant.

Supplementary Figure 5

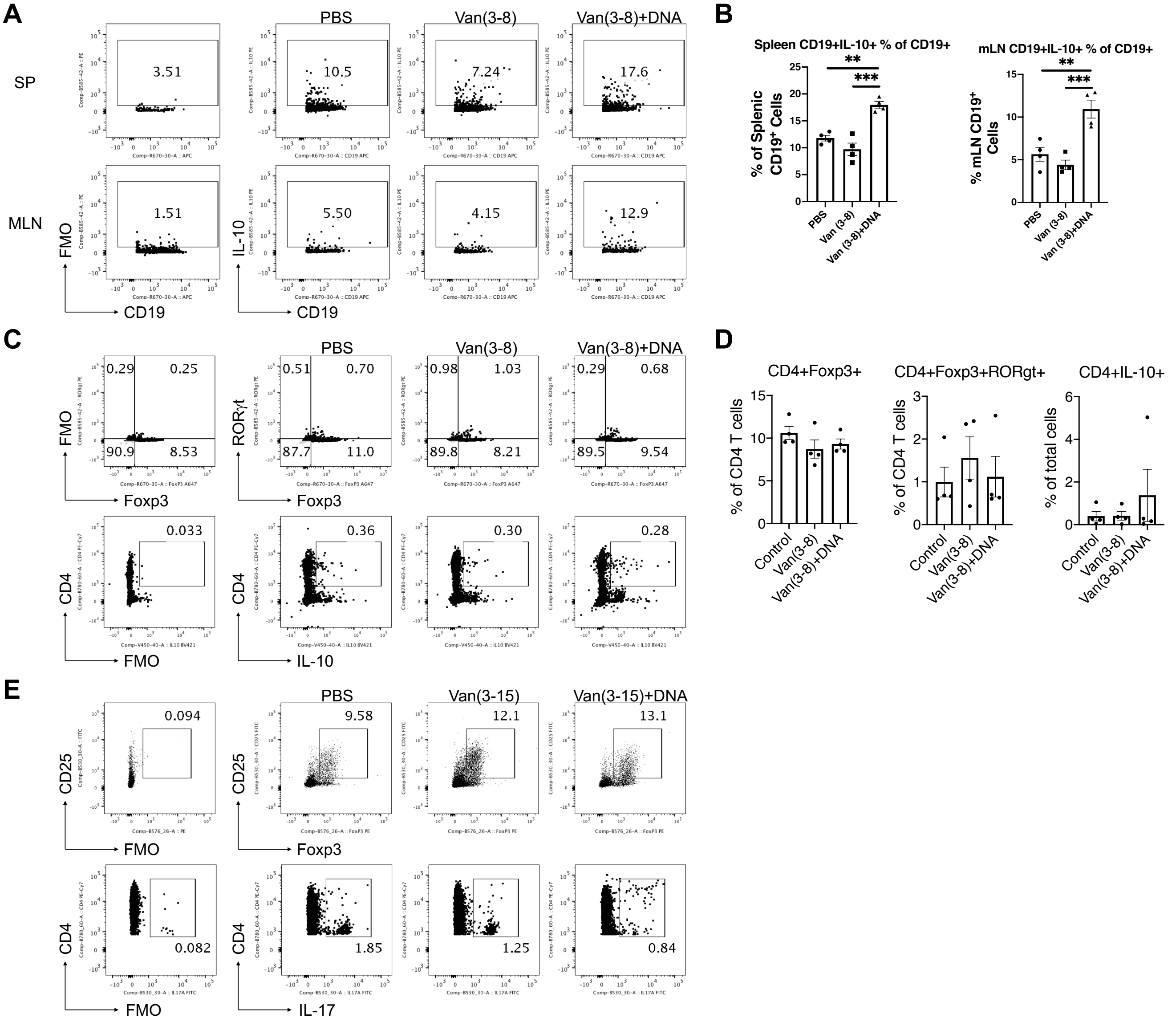

Figure S5. (A) Representative FACS plots of IL-10 producing Breg cells in the spleen (SP) and mesenteric lymph node (MLN) at 8 weeks of age. Graphs were pre-gated on B cells. FMO, fluorescence minus one. (B) Percentage of IL-10 producing Breg cells in total B cells in the spleen and MLN at 8 weeks of age. **p<0.01, ***p<0.001. (C) Representative FACS plots of Treg cells and IL-10 producing cells in the MLN at 8 weeks of age. Upper graphs were pre-gated on $CD4^+$ cells. (D) Percentage of $Foxp3^+$ Treg cells, $Foxp3^+ROR\gamma t^+$ Treg cells, and IL-10 producing $CD4^+$ T cells in the MLN at 8 weeks of age. (E) Representative FACS plots of Treg and IL-17 producing $CD4^+$ cells in the spleen at 15 weeks of age. Graphs were pre-gated on $CD4^+$ cells.

Supplementary Figure 6

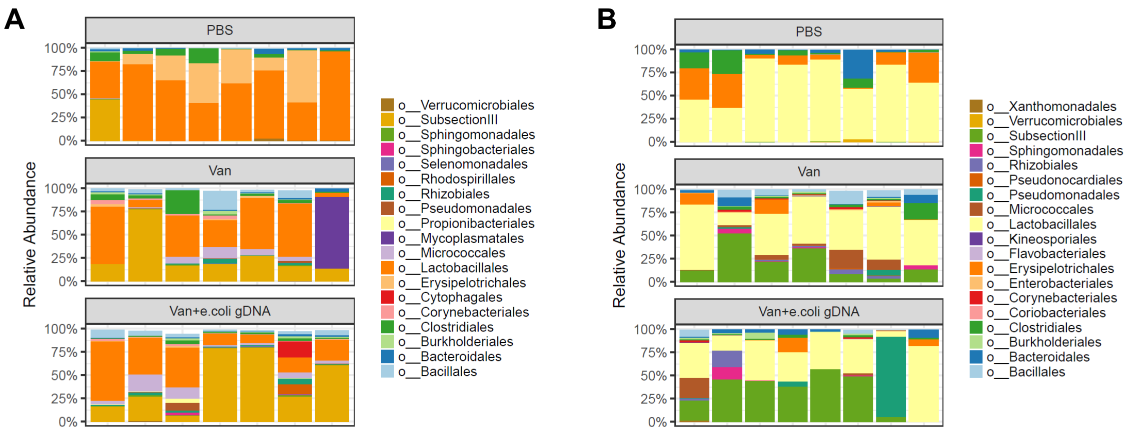

Figure S6. Relative abundance of bacteria at order level in the duodenum/jejunum contents (A) or Ileum contents (B) at 8 weeks of age (n≥7).

Supplemental Table I. Primer sequences used for RT-qPCR analysis.

Primer Name	Primer Sequence Forward (5’-3’)	Primer Sequence Reverse (5’-3’)
L32	GCCAGGAGACGACAAAAAT	AATCCTCTTGCCCTGATCC
IL-17A	ATCCCTCAAAGCTCAGCGTGTC	GGGTCTTCATTGCGGTGGAGAG
Has1	CAGAGCCTCTTCGCTTACCT	TAGGCTGAGATGGTGAGTGC
Has2	TCCTCAGCAGCGTGAGATAC	TTCATGCAGCAAGGAGTTTC
Has3	ACTGCCTTCAAGGCCCTTGG	AATGTTCCAGATGCGGCCAC
IL-1β	TGGACCTTCCAGGATGAGGACA	GTTCATCTCGGAGCCTGTAGTG
TNFα	GGTGCCTATGTCTCAGCCTCTT	GCCATAGAACTGATGAGAGGGAG
IL-6	CTCTGGGAAATCGTGGAAAT	CCAGTTTGGTAGCATCCATC
